# In vivo mutagenicity assessment of orally treated *tert*-butyl hydroperoxide in the liver and glandular stomach of MutaMouse

**DOI:** 10.1186/s41021-023-00285-2

**Published:** 2023-11-21

**Authors:** Yasumasa Murata, Kenichiro Suzuki, Yoshiyuki Shigeta, Takako Iso, Nozomu Hirose, Takaaki Umano, Katsuyoshi Horibata, Kei-ichi Sugiyama, Akihiko Hirose, Kenichi Masumura, Mariko Matsumoto

**Affiliations:** 1 Division of Risk Assessment, National Institute of Health Sciences, Kanagawa, Japan; 2 Genotoxicology Laboratory, BioSafety Research Center Inc., Shizuoka, Japan; 3 Division of Chemical Information, National Institute of Occupational Safety and Health, Kanagawa, Japan; 4 Division of Genetics and Mutagenesis, National Institute of Health Sciences, Kanagawa, Japan; 5 Chemicals Evaluation and Research Institute, Tokyo, Japan

**Keywords:** TG488, MutaMouse, TBHP, In vivo mutagenicity, Transgenic rodent gene mutation assay

## Abstract

**Background:**

*tert*-Butyl hydroperoxide (TBHP; CAS 75–91-2), a hydroperoxide, is mainly used as a polymerization initiator to produce polyethylene, polyvinyl chloride, and unsaturated polyester. It is a high-production chemical, widely used in industrial countries, including Japan. TBHP is also used as an additive for the manufacturing of food utensils, containers, and packaging (UCP). Therefore, there could be consumer exposure through oral intake of TBHP eluted from UCPs. TBHP was investigated in various in vitro and in vivo genotoxicity assays. In Ames tests, some positive results were reported with and/or without metabolic activation. As for the mouse lymphoma assay, the positive result was reported, regardless of the presence or absence of metabolic activation enzymes. The results of some chromosomal aberrations test and comet assay in vitro also demonstrated the genotoxic positive results. On the other hand, in in vivo tests, there are negative results in the bone marrow micronucleus test of TBHP-administered mice by single intravenous injection and the bone marrow chromosomal aberration test using rats exposed to TBHP for 5 days by inhalation. Also, about dominant lethal tests, the genotoxic positive results appeared. In contrast, there is little information about in vivo mutagenicity and no information about carcinogenicity by oral exposure.

**Results:**

We conducted in vivo gene mutation assay using MutaMice according to the OECD Guidelines for the Testing of Chemicals No. 488 to investigate in vivo mutagenicity of TBHP through oral exposure. After repeated dosing for 28 days, there were no significant differences in the mutant frequencies (MFs) of the liver and glandular stomach up to 300 mg/kg/day (close to the maximum tolerable dose (MTD)). The positive and negative controls produced the expected responses.

**Conclusions:**

These findings show that orally administrated TBHP is not mutagenic in the mouse liver and glandular stomach under these experimental conditions.

**Supplementary Information:**

The online version contains supplementary material available at 10.1186/s41021-023-00285-2.

## Background

*tert*-Butyl hydroperoxide (TBHP; CAS 75-91-2) is mainly used as a polymerization initiator to produce polyethylene, polyvinyl chloride, and unsaturated polyester. It is a high-production chemical widely used in industrial countries, including Japan, and is produced from the direct reaction of isobutane and liquid oxygen, or from tertiary butyl alcohol and hydrogen peroxide in the presence of sulfuric acid [1, 2]. TBHP is also used as an additive for the manufacture of food utensils, containers, and packaging (UCP) and one of the chemicals listed on a positive list (PL) of the “Food Sanitation Act” in Japan. Therefore, there could be consumer exposure through oral intake of TBHP eluted from UCPs.

In order to assess the risk of the listed chemicals on the PL, we preliminary evaluated the genotoxicity of TBHP. Quantitative structure–activity relationship (QSAR) predictions (Derek Nexus ver.6.0.1, Lhasa Limited, UK, and CASE Ultra ver.1.6.2.2, MultiCASE Inc., USA) for bacterial gene mutation assays (Ames test) [3] indicated positive for TBHP. Organic hydroperoxides, including TBHP, are commonly used as a model compound to study oxidative cell injury and DNA damage caused by their high reactivity [4–9]. Regarding in vitro assays, in Ames tests, some positive results were reported with and/or without metabolic activation were reported [5, 6, 10–19]. In the comet assay in vitro, the positive results have been reported by using cultured cells [4, 7–9]. The result of the micronucleus test in vitro also demonstrated genotoxicity as positive [20]. In contrast, in the in vivo assay, DNA adducts were detected in the liver and stomach of male Wistar rats exposed to TBHP by single gavage administration, suggesting genotoxicity [21]. In addition, in terms of dominant lethal assays, positive genotoxic results appeared by i.p. injection [22, 23], whereas there was also a negative result in the comet assay using rats administered a single subcutaneous injection of TBHP [24] and in the bone marrow micronucleus test of mice by single intravenous injection [2]. TBHP is known to rapidly metabolize into 2-methylpropan-2-ol intravitally [21], and it has been reported not to be genotoxic in most in vitro and in vivo studies [25].

For the carcinogenicity test, there are no results for inhalation and oral exposure to TBHP. The carcinogenicity of 2-methylpropan-2-ol, the main metabolite of TBHP, by oral administration demonstrated a small increase in systemic adenomas in mice and rats [25–27]. The dose level of adenomas produced by 2-methylpropane-2-ol is higher than the LD_50_ of TBHP, suggesting that chronic exposure to TBHP probably will not cause the dose level of 2-methylpropane-2-ol-inducing systemic tumors [22]. TBHP is mutagenic in in vitro assays, so carcinogenesis may occur at the site of direct contact. However, because TBHP is rapidly metabolized, it is unlikely to reach various organs by inhalation and oral administration.

The potential for local carcinogenicity of TBHP has rarely been investigated by practical assays, and few studies on dermal exposure were reported [28]. After dermal application, TBHP carcinogenicity was not observed. However, TBHP, but not 2-methylpropan-2-ol, promoted dermal tumor development after induction by 4-nitroquinoline1-oxide [29]. Previous studies suggest that TBHP is genotoxic and mutagenic in vitro, and probably genotoxic in vivo. However, mutagenicity in vivo remains ambiguous. Carcinogenesis through the relevant pathway is likely to be restricted to somatic cells in the tissue of initial contact, and this may result in local carcinogenicity. For further verification, we conducted the in vivo gene mutation assay of TBHP in the liver and glandular stomach (the tissues of first contact), using MutaMice to investigate in vivo mutagenicity of TBHP through oral exposure.

## Results

### Treatment and general observations

**Fig. 1:**
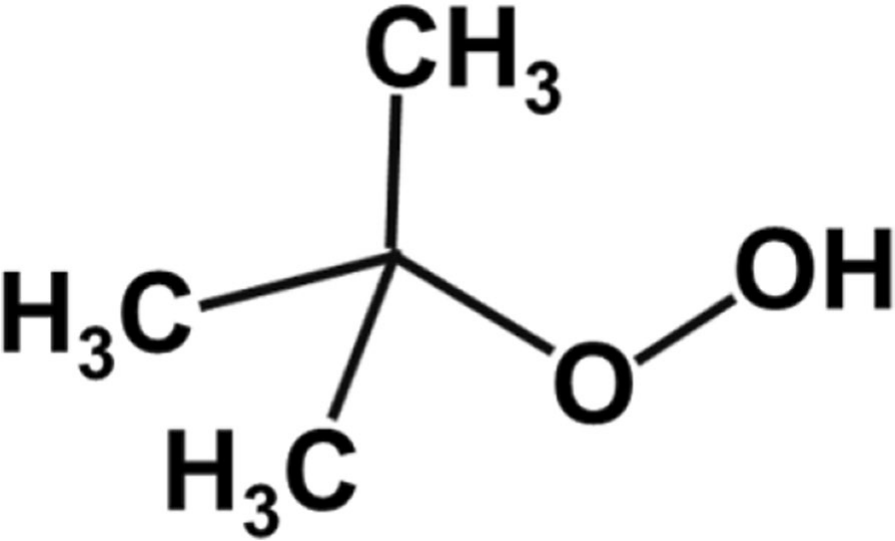
*tert*-Butyl hydroperoxide structural formula

Transgenic rodent gene mutation assay was carried out according to the OECD Guidelines for the Testing of Chemicals No. 488. Based on the results of the dose-finding study, the male MutaMice were orally administrated with TBHP at 75, 150, and 300 mg/kg/day for 28 days and liver and glandular stomach were collected 3 days after the final treatment for gene mutation assay. During the dosing period, in the TBHP treatment group of 150 and 300 mg/kg/day, all individuals demonstrated an increase in locomotor activity after administration on day 22 and later (Supplementary Table 1). All other dose groups did not show variation in the general conditions. Body weights did not differ significantly from those of the control group (Fig. 1). In gross pathological examination, there were no grave pathological findings in both the TBHP treatment group and the control group.

### Mutation assay

Genomic DNA was extracted from the tissues and *lacZ* mutation assay was conducted. In the liver, the average value ± standard deviation of the *lacZ* MF was 47.2 ± 10.6 (× 10^−6^) in a negative control group, while in TBHP-treated groups, the MFs were 45.8 ± 13.1 (× 10^−6^), 42.5 ± 10.5 (× 10^−6^), and 42.7 ± 8.2 (× 10^−6^) in 75, 150, and 300 mg/kg/day treatment group, respectively (Table 1). These TBHP-treated values were not significantly different from the values in the negative control group (75 mg/kg/day treatment group vs. negative control: *p* = 0.9939, 150 mg/kg/day treatment group vs. the negative control: *p* = 0.8345, 300 mg/kg/day treatment group vs. the negative control: *p* = 0.8460, Dunnett test). These MFs were not dose dependent and within the range of historical negative control data (mean ± S.D. = 43.1 ± 13.5, Supplementary Table 2). The average of MF In the positive control group was 183.5 ± 14.3 (× 10^−6^), indicating a significant increase from the negative control group (*p* = 0.0001, Student’s t-test).

**Table 1:** Mutant frequencies in the livers of MutaMouse given TBHP. Male mice were dosed once daily for 28 days (oral administration, tissues were collected 3 days after final administration)

Substance	Dose (mg/kg/day, p.o.)	Animal ID No	Number of plaque	Number of packaging	Number of mutants	Mutant frequency (× 10^−6^)	Group Mean ± S.D. (× 10^−6^)
0.5% MC	0	3001	666,900	1	32	48.0	47.2 ± 10.6
		3002	725,400	1	22	30.3	
		3003	635,400	1	35	55.1	
		3004	965,700	1	44	45.6	
		3005	1,071,900	1	61	56.9	
TBHP	75	3101	945,900	1	33	34.9	45.8 ± 13.1
		3102	800,100	1	30	37.5	
		3103	395,100	1	26	65.8	
		3104	1,126,800	1	59	52.4	
		3105	990,000	1	38	38.4	
	150	3201	676,800	1	26	38.4	42.5 ± 10.5
		3202	904,500	1	45	49.8	
		3203	1,066,500	1	60	56.3	
		3204	642,600	1	19	29.6	
		3205	1,348,200	1	52	38.6	
	300	3301	736,200	1	29	39.4	42.7 ± 8.2
		3302	981,900	1	54	55.0	
		3303	1,429,200	1	50	35.0	
		3304	771,300	1	36	46.7	
		3305	1,126,800	1	42	37.3	
ENU	100	3401	559,800	1	107	191.1	183.5 ± 14.3 *
		3402	920,700	1	176	191.2	
		3403	1,164,600	1	215	184.6	
		3404	649,800	1	103	158.5	
		3405	582,300	1	112	192.3	

0.5% MC: Control group (0.5 w/v% methylcellulose solution, 10 ml/kg)

ENU: Positive control (*N*-ethyl-*N*-nitrosourea, 10 ml/kg, i.p., once daily for 2 days, expression period; 10 days)

^*^Statistically significant difference from the negative control (Student’s *t*-test: *p* < 0.05)

In the glandular stomach, the average value ± standard deviation of the *lacZ* MFs was 44.2 ± 9.0 (× 10^−6^) in the negative control group, while in TBHP-treated groups, the MFs were 41.0 ± 11.6 (× 10^−6^), 42.5 ± 11.1 (× 10^−6^), and 46.3 ± 7.0 (× 10^−6^) in 75, 150, and 300 mg/kg/day treatment group, respectively (Table 2). These TBHP-treated values were not significantly different from the values in the negative control group (75 mg/kg/day treatment group vs. the negative control: *p* = 0.9223, 150 mg/kg/day treatment group vs. the negative control: *p* = 0.9857, 300 mg/kg/day treatment group vs. negative control: *p* = 0.9739, Dunnett test). These MFs were not dose-dependent and within the range of historical negative control data (mean ± S.D. = 43.8 ± 12.8, Supplementary table).

**Table 2:** Mutant frequencies in the glandular stomach of MutaMouse given TBHP. Male mice were dosed once daily for 28 days (oral administration, tissues were collected 3 days after final administration)

Substance	Dose (mg/kg/day, p.o.)	Animal ID No	Number of plaque	Number of packaging	Number of mutants	Mutant frequency (× 10^−6^)	Group Mean ± S.D. (× 10^−6^)
0.5% MC	0	3001	1,215,900	1	54	44.4	44.2 ± 9.0
		3002	575,100	1	32	55.6	
		3003	890,100	1	28	31.5	
		3004	908,100	1	37	40.7	
		3005	739,800	1	36	48.7	
TBHP	75	3101	1,323,900	1	46	34.7	41.0 ± 11.6
		3102	891,900	1	26	29.2	
		3103	669,600	1	23	34.3	
		3104	888,300	1	46	51.8	
		3105	1,015,200	1	56	55.2	
	150	3201	497,700	1	16	32.1	42.5 ± 11.1
		3202	1,053,000	1	49	46.5	
		3203	886,500	1	30	33.8	
		3204	724,500	1	43	59.4	
		3205	887,400	1	36	40.6	
	300	3301	828,000	1	40	48.3	46.3 ± 7.0
		3302	1,122,300	1	50	44.6	
		3303	1,343,700	1	58	43.2	
		3304	1,100,700	1	42	38.2	
		3305	1,086,300	1	62	57.1	
ENU	100	3401	957,600	1	375	391.6	410.9 ± 20.5 *
		3402	1,125,000	1	490	435.6	
		3403	1,607,400	1	647	402.5	
		3404	1,238,400	1	489	394.9	
		3405	916,200	1	394	430.0	

0.5% MC: Control group (0.5 w/v% methylcellulose solution, 10 ml/kg)

ENU: Positive control (*N*-ethyl-*N*-nitrosourea, 10 ml/kg, i.p., once daily for 2 days, expression period: 10 days)

^*^Significant difference from the negative control (Student's test: *p* < 0.05)

The average of MF in the positive control group was 410.9 ± 20.5 (× 10^−6^), indicating a significant increase from the negative control group (*p* = 0.0001, Student’s t-test).

## Discussion

**Fig. 2:**
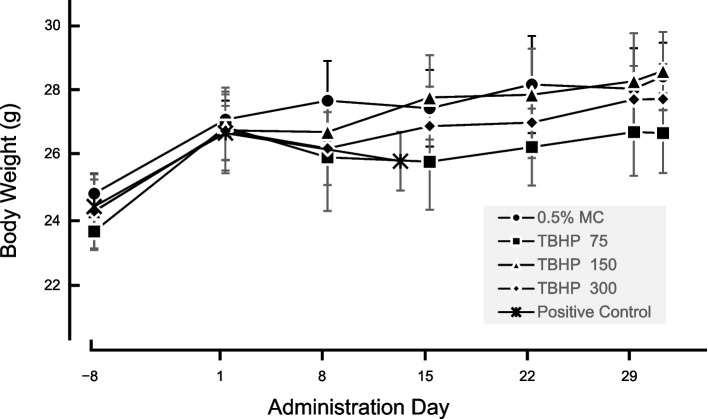
MutaMouse body weight over a 28-day administration period (after day 1) from the day of delivery (day − 8). Each graph depicts the average weight value ± standard deviations (6 animals in negative control, TBHP 75, TBHP 150, and positive control groups, 8 animals in TBHP 300 mg/kg/day group.)

The 28-days repeated dosing study showed no deaths in mice treated with TBHP up to the highest dose, 300 mg/kg/day. In terms of the general condition, the administration of TBHP induced an increase in locomotor activity in all individuals in treatment groups of 150 and 300 mg/kg/day from the 22^nd^ day of the experiment. In other treatment groups, no observed changes in clinical symptoms were observed. Body weight was never affected by TBHP treatment (Fig. 2), and, in addition, there were no gross pathological changes in the extracted liver and glandular stomach.

The treatment groups did not show a significant increase in MF values in the liver and glandular stomach of MutaMouse treated with TBHP. All MFs in the treatment and negative control groups were within the range of historical negative control data (Supplementary table). Consequently, the mutagenicity of TBHP was demonstrated to be negative in the liver and glandular stomach of MutaMouse in this study.

Although 1000 mg/kg/day has been shown to exceed the MTD in the dose-finding study (see Methods), at the highest dose in the main experiment, 300 mg/kg/day, no significant effect on body weight nor gross pathological findings was observed. Meanwhile, locomotor activity increased in all treated individuals of dose groups greater than 150 mg/kg/day. Recently, Saha et al. (2019) proved the related matters between the damaged kidney exposed to TBHP and the stress and impact on the brain in mice, leading the behavioral anomalies [30]. It is possible that this crosslink of behavioral anomalies and TBHP toxicity might be one of the causes of the locomotor activity in our evaluation.

Furthermore, a previously reported subacute (14-day exposure) oral toxicity study in mice demonstrated that forestomach inflammation was occurred by the gavage administration of 176 and 352 mg/kg. Histopathological findings found forestomach hyperplasia in tissue slice samples of 88 mg/kg and above doses [1]. In contrast, in our study, tissue samples were collected 3 days after the final treatment of 28 days of repeated dosing. The histopathological examination was not conducted. Therefore, although we cannot make a simple comparison of these results, these reports strongly suggested that the target organs of this study, the glandular stomach and liver, were properly exposed to test substances by oral gavage administration of TBHP. Furthermore, 1,000 mg/kg was the lethal dose in the dose-finding test, and then we followed the procedure of the Guidelines for the Testing of Chemicals No. 488, setting the geometric ratio at three.

These imply that the dose-setting of doses, the maximum dose of 300 mg/kg/day, could be appropriate for this assay. In the investigation of absorption and metabolic disposition using radiolabeled [^14^C]-TBHP, a single gavage exposure of [^14^C]-TBHP to male rats at 10 and 100 mg/kg was well absorbed and induced the maximum blood concentration of ^14^C at 9.2 and 68.5 µg/ml, respectively [2]. The radioactivity concentration of [^14^C] at 48 h after oral [^14^C]-TBHP administration, was highest in the kidneys, followed by the liver and blood. These previous results suggest that the TBHP administered orally and/or its metabolites reached the liver in this study.

Despite the negative result in mutagenicity in this study, most of the previous results of the mutagenicity test in vitro denoted positive results, which are supposed to be induced by high oxidative damage [10–13, 15–18, 31–34]. Previously, some Ames tests of TBHP resulted in positive results with or without the S9 mix [10–19], and additionally, it was also revealed that the positive response of TBHP on the Ames test was weakened along with the dose of antioxidant addition[35]. TBHP is known to be metabolized to free radical intermediates by cytochrome P450, and the oxygen free radicals (Reactive oxygen species: ROS) induce intravital cellular changes that cause DNA damage [36–40]. However, TBHP was also reported to convert rapidly converted in vivo, mainly to 2-methylpropan-2-ol (CAS 75-65-0) [21], which was tested for mutagenicity by the NTP in 1995 and all in vitro and in vivo results were negative [25, 41]. In particular, in liver tissue, abundant endogenous antioxidants can scavenge ROSs [42]. Therefore, the scavenging activities are considered to be one of the reasons for the negative results of in vivo mutagenicity of TBHP. Some other tertiary hydroperoxides have structures similar to TBHP, cumene hydroperoxide (CHP, CAS 80–15-9), and *tert*-amyl hydroperoxide (TAHP, CAS 3425–61-4). Both substances demonstrated positive and equivocal results in bacterial mutagenicity assays [43, 44]. Generally, hydroperoxides are known to be highly reactive and activate metabolism [21, 45]. Due to their high reactivity, they tend to be positive in vitro while in vivo they are rapidly deoxidized by glutathione, one of the radical scavengers that are typically found in living bodies among a wide range of organisms [46], with the reduction of hydrogen peroxidase coupling to oxidation of glucose 6-phosphate [47], and 2-methylpropan-2-ol is produced in the case of TBHP destruction [21]; thus it may not be mutagenic in most intravital cases.

## Conclusions

We conducted the in vivo gene mutation assay of TBHP in the liver and glandular stomach using MutaMice. In both organs, no significant increase in MFs was observed in the treated groups up to 300 mg/kg/day (close to MTD). Consequently, the mutagenicity of TBHP was determined to be negative in the liver and glandular stomach of MutaMice in this experimental condition. This study suggested that TBHP is not mutagenic in vivo with oral exposure. For better regulation of safety and manufacturing, these results are worthwhile in evaluating the risk of carcinogenicity of TBHP.

## Methods

The transgenic rodent gene mutation assay was carried out according to the OECD Guidelines for the Testing of Chemicals No. 488. (26 June 2020: Transgenic rodent somatic and Germ Cell Gene Mutation Assays). The experiments were conducted at the BioSafety Research Center (BSRC: Shizuoka, Japan). According to “the Act on Animal Welfare and Management,” “the standards relating to the care and management of laboratory animals and pain relief.” and “BSRC Guidelines for Animal Experimentation.” The animals were cared for according to the “Act on the Conservation and Sustainable Use of Biological Diversity through Regulations on the Use of Living Modified Organisms,” and the “BSRC Safety Management Regulations for the Recombinant DNA Experiment.”

### Chemicals

Tokyo Chemical Industry Co., Ltd. (Tokyo) supplied TBHP (CAS: 75-91-2, purity: 70.7%) and P-gal (phenyl-β-d-galactoside) (CAS: 2818-58-8). Kanto Chemical Co., Inc. supplied disodium hydrogenphosphate (CAS: 10039-32-4), NaCl (CAS: 7647-14-5), KCl (CAS: 7447-40-7), and sucrose. *N*-ethyl-*N*-nitrosourea (ENU), a positive control substance, was purchased from Toronto Research Chemicals Inc. (Ontario, Canada). Next, 0.5 w/v% MC400 solution, Proteinase K, SDS, and Potassium Dihydrogen Phosphate were purchased from FUJIFILM Wako Pure Chemical Corp. (Osaka). EDTA and RNase A were purchased from Nippon Gene Co., Ltd. (Tokyo, Japan).

### Animals and treatment

Male and female CD2F1 mice, as well as a male MutaMice, were obtained from Japan SLC, Inc. (Shizuoka, Japan) and Transgenic Inc. (Kobe, Japan), respectively. After a 6-day acclimatization period, 12 male and female animals found to be in good health were used in the dose-finding study, and after an 8-day acclimatization period, 32 male MutaMice were similarly selected for use in the main study. In the dose-finding study, three animals were treated for each dose in both males and females. As there were no differences in the toxicity induced by oral administration of TBHP between males and females in the dose-finding study, only male individuals were treated in the main study according to the OECD TG 488, as our previous report [48]. These animals were reared on a basal diet, CRF-1 (Oriental yeast), and water ad libitum. The animals were kept at a room temperature of 20 °C to 26 °C, relative humidity of 35% to 70%, a 12 h light/dark cycle, and 12 air changes per hour. Groups of three CD2F1 mice/sex were administered TBHP by gavage once a day for 2 weeks for a dose-finding study in a volume of 10 ml/kg, and at 30.0, 100, 300, and 1,000 mg/kg of purity-corrected TBHP. The highest dose level was established based on the OECD Guidelines for the Testing of Chemicals No. 488, and the total four levels were divided by the geometric ratio 3. Both males and females treated with 1,000 mg/kg/day all died on day 2. Furthermore, there was no variation in condition or body weight in the treatment groups of 30, 100, and 300 mg/kg/day. Based on the results of the dose-finding study, we considered 75, 150, and 300 mg/kg/day doses of purity-corrected TBHP to be used for 28-day repeated administration. For the main study, the vehicle control (0.5%MC) group was maintained similarly. The positive control was treated with ENU (i.p.) at 100 mg/kg/day once a day for 2 days. The euthanasia of the experimental animals and the extraction of the target organs were carried out 3 days after the last administration in the TBHP-treated and negative control group, and 10 days after that in the positive control group. Six animals were treated in all groups (only in the case of the 300 mg/kg/day group, eight animals were treated). The animals were observed once a day every day. Body weight was recorded on the administration days 1, 8, 15, and 22, and 1 and 3 days after the last treatment. We selected each of the five animal samples in the ascending order of animal ID for mutation analysis.

The liver and glandular stomach were collected after the euthanasia using carbon dioxide gas, and a gross pathological examination was performed. In the liver, two points of the left lateral lobe were hollowed out and were frozen by liquid N_2_ (LN_2_) in each microtube. The remaining lobes and another lobe packed in a plastic bag were crushed and frozen in a flat bottom metal container filled with LN_2_. Additionally, part of the glandular stomach was divided and frozen with LN_2_ in a laboratory storage bag. The remaining part of the forestomach was disposed of. Frozen samples were stored in an ultra-deep freezer (set temperature: − 80 °C; standard value: − 90 °C to − 60 °C) until analysis. Five animal tissue samples were analyzed in each group of mutation assay.

### DNA isolation

The following procedures were used to extract genomic DNA from the liver and glandular stomach [49]. The frozen tissue was homogenized by a pestle with the Dounce buffer (1.7 g of N_2_HPO_4_, 0.25 g of KH_2_PO_4_, 8.0 g NaCl, 0.20 g of KCl, and 20 ml of 0.5 mol/L EDTA in 1000 ml of water) in a Dounce homogenizer. The homogeneous mixture was poured into an ice-cold centrifuge tube containing a 0.5 mol/L sucrose in Dounce buffer. The supernatant was removed after centrifugation at 3,000 r/min (1750 G) for 10 min. The precipitated nuclei/cells were suspended with 3 ml of RNase (prepared by 100 ml of Dounce buffer and 2.0 ml of RNase A (10 mg/ml)) and mixed with 3 ml of proteinase K solution (prepared with 200 mg proteinase K, 60 ml of distilled water, 20 ml of 10 w/v% SDS solution, and 20 ml of 0.5 mol/L of EDTA adjusted at pH 7.5), followed by incubation at 50 °C for 2–2.5 h. A (1:1) mixture of phenol and chloroform was added, and the water layer was separated after 10 min of centrifugation at 2,500 r/min (1220 G). Similarly, chloroform/isoamyl alcohol (24:1) and the water layer were mixed and centrifuged. The water layer was added to another centrifuge tube, and ethanol was added to precipitate the DNA. DNA was soaked in 70% ethanol for 10 min. After ethanol evaporation, the DNA was dissolved in a TE buffer (NIPPON GENE) at room temperature overnight. The DNA solution was placed in the refrigerator at 4 °C. The NanoDrop (AGC TECHNO GLASS Co., Ltd. (Shizuoka, Japan)) was used to determine the concentration of DNA.

### In vitro packaging

The Lambda in vitro packaging reaction was carried out for transgene rescue according to the Transpack instruction manual (Agilent Technologies, Transpack Packaging Extract Catalog #200220, #200221, and #200223). Approximately 10 µl of the genomic DNA solution (100–600 μg/ml) was gently mixed with the dedicated Transpack packaging tube and incubated at 30 °C for 1.5 h twice before being mixed with 700 µl of SM buffer.

### Mutant frequency determination

A mixture of 2 ml of *Escherichia coli* C strain (*lacZ*^*−*^*, gal E*^*−*^) suspension and the whole amount of packaged sample, ~ 700 µl volume, were stirred and incubated for 30 min, and the rescued phages were absorbed into *E. coli*. This solution was diluted ten times by adding 30 µl of solution to 270 µl of LB culture medium containing 10 mmol/L of magnesium sulfate. Then 30 µl of this dilution was mixed with the *E. coli* suspension in the titer tube, and an aliquot of this suspension was mixed with LB top agar for the titer plates. The remaining cell suspension was mixed with LB top agar containing P-gal (phenyl-β-d-galactoside) for the selection plates. Both plates were incubated overnight at 37 °C. These packaging procedures were performed once since the total number of plaques was reached 300,000. The total number of plaques (*N*) was calculated by the following formula, using the total number of plaques on the titer plate (*n*).

$$N=\frac{n \times 300 \,\left(\mathrm{\mu l}\right)\times 2700\, \left(\mathrm{\mu l}\right)}{30 \,\left(\mathrm{\mu l}\right) \times 30\, \left(\mathrm{\mu l}\right)}$$

The MF was calculated as follows: MF = the total number of plaques on the selection plates (*s*) divided by the total number of plaques (*N*).

$$MF=\frac{s}{N}$$

### Statistical analysis

The Bartlett test was used to assess data homogeneity in the treatment and negative control groups. When the homogeneity was detected, the Dunnett test was used to analyze the data. For nonhomogeneous data, Steel’s test was used. Based on the result of F-test, the Student’s *t*-test or Aspin–Welch's *t*-test was used to compare MFs between negative and positive controls. The significance criterion was set at 5% probability levels.

### Supplementary Information

**Additional file 1: Supplementary Table 1.** Clinical observations in the gene mutation assay of *tert*-butyl hydroperoxide. **Supplementary Table 2.** Historical data of negative controls (Transgenic rodent gene mutation assay (*lacZ* assay)).

## Data Availability

All data generated or analysed during this study are included in this published article.

## Abbreviations

TBHP: *tert*-Butyl hydroperoxide
UCP: Food utensils, containers, and packaging
MTD: Maximum tolerable dose
PL: Positive list
QSAR: Quantitative structure–activity relationship
MF: Mutant frequency
ROS: Reactive oxygen species
CHP: Cumene hydroperoxide;
TAHP: *tert*-Amyl hydroperoxide
BSRC: BioSafety Research Center
ENU: *N*-Ethyl-*N*-nitrosourea
LN_2_: Liquid N_2_
