# Supplementary material for: In vivo mutagenicity assessment of orally treated tert-butyl hydroperoxide in the liver and glandular stomach of MutaMouse
Source: Genes Environ. 2023 Nov 21;45:29. doi: 10.1186/s41021-023-00285-2 (PMC10662197; doi:10.1186/s41021-023-00285-2)
Supplement: Supplementary file 1 — Additional file 1: Supplementary Table 1. Clinical observations in the gene mutation assay of tert-butyl hydroperoxide. Supplementary Table 2. Historical data of negative controls (Transgenic rodent gene mutation assay (lacZ assay)). [file 41021_2023_285_MOESM1_ESM.docx]

Supplementary table 1. Clinical observations in the gene mutation assay of *tert*-butyl hydroperoxide

|  |  |  |  | Day of experiment | | | | | | | | | | | | | |
| --- | --- | --- | --- | --- | --- | --- | --- | --- | --- | --- | --- | --- | --- | --- | --- | --- | --- |
|  | Substance | Dose (mg/kg/day, p.o.) | Animal ID No. | 1 | | 2 | | 3 | | 4 | | 5 | | 6 | | 7 | |
|  |  |  |  | 1st | 2nd | 1st | 2nd | 1st | 2nd | 1st | 2nd | 1st | 2nd | 1st | 2nd | 1st | 2nd |
|  | 0.5% MC | 0 | 3001 | N | N | N | N | N | N | N | N | N | N | N | N | N | N |
|  |  |  | 3002 | N | N | N | N | N | N | N | N | N | N | N | N | N | N |
|  |  |  | 3003 | N | N | N | N | N | N | N | N | N | N | N | N | N | N |
|  |  |  | 3004 | N | N | N | N | N | N | N | N | N | N | N | N | N | N |
|  |  |  | 3005 | N | N | N | N | N | N | N | N | N | N | N | N | N | N |
|  |  |  | 3006 | N | N | N | N | N | N | N | N | N | N | N | N | N | N |
|  | TBHP | 75 | 3101 | N | N | N | N | N | N | N | N | N | N | N | N | N | N |
|  |  |  | 3102 | N | N | N | N | N | N | N | N | N | N | N | N | N | N |
|  |  |  | 3103 | N | N | N | N | N | N | N | N | N | N | N | N | N | N |
|  |  |  | 3104 | N | N | N | N | N | N | N | N | N | N | N | N | N | N |
|  |  |  | 3105 | N | N | N | N | N | N | N | N | N | N | N | N | N | N |
|  |  |  | 3106 | N | N | N | N | N | N | N | N | N | N | N | N | N | N |
|  |  | 150 | 3201 | N | N | N | N | N | N | N | N | N | N | N | N | N | N |
|  |  |  | 3202 | N | N | N | N | N | N | N | N | N | N | N | N | N | N |
|  |  |  | 3203 | N | N | N | N | N | N | N | N | N | N | N | N | N | N |
|  |  |  | 3204 | N | N | N | N | N | N | N | N | N | N | N | N | N | N |
|  |  |  | 3205 | N | N | N | N | N | N | N | N | N | N | N | N | N | N |
|  |  |  | 3206 | N | N | N | N | N | N | N | N | N | N | N | N | N | N |
|  |  | 300 | 3301 | N | N | N | N | N | N | N | N | N | N | N | N | N | N |
|  |  |  | 3302 | N | N | N | N | N | N | N | N | N | N | N | N | N | N |
|  |  |  | 3303 | N | N | N | N | N | N | N | N | N | N | N | N | N | N |
|  |  |  | 3304 | N | N | N | N | N | N | N | N | N | N | N | N | N | N |
|  |  |  | 3305 | N | N | N | N | N | N | N | N | N | N | N | N | N | N |
|  |  |  | 3306 | N | N | N | N | N | N | N | N | N | N | N | N | N | N |
|  |  |  | 3307 | N | N | N | N | N | N | N | N | N | N | N | N | N | N |
|  |  |  | 3308 | N | N | N | N | N | N | N | N | N | N | N | N | N | N |
|  | ENU | 100 | 3401 | N | | N | N | N | N | N | | N | | N | | N | |
|  |  |  | 3402 | N | | N | N | N | N | N | | N | | N | | N | |
|  |  |  | 3403 | N | | N | N | N | N | N | | N | | N | | N | |
|  |  |  | 3404 | N | | N | N | N | N | N | | N | | N | | N | |
|  |  |  | 3405 | N | | N | N | N | N | N | | N | | N | | N | |
|  |  |  | 3406 | N | | N | N | N | N | N | | N | | N | | N | |
|  |  |  |  | Day of experiment | | | | | | | | | | | | | |
|  | Substance | Dose (mg/kg/day, p.o.) | Animal ID No. | 8 | | 9 | | 10 | | 11 | | 12 | | 13 | | 14 | |
|  |  |  |  | 1st | 2nd | 1st | 2nd | 1st | 2nd | 1st | 2nd | 1st | 2nd | 1st | 2nd | 1st | 2nd |
|  | 0.5% MC | 0 | 3001 | N | N | N | N | N | N | N | N | N | N | N | N | N | N |
|  |  |  | 3002 | N | N | N | N | N | N | N | N | N | N | N | N | N | N |
|  |  |  | 3003 | N | N | N | N | N | N | N | N | N | N | N | N | N | N |
|  |  |  | 3004 | N | N | N | N | N | N | N | N | N | N | N | N | N | N |
|  |  |  | 3005 | N | N | N | N | N | N | N | N | N | N | N | N | N | N |
|  |  |  | 3006 | N | N | N | N | N | N | N | N | N | N | N | N | N | N |
|  | TBHP | 75 | 3101 | N | N | N | N | N | N | N | N | N | N | N | N | N | N |
|  |  |  | 3102 | N | N | N | N | N | N | N | N | N | N | N | N | N | N |
|  |  |  | 3103 | N | N | N | N | N | N | N | N | N | N | N | N | N | N |
|  |  |  | 3104 | N | N | N | N | N | N | N | N | N | N | N | N | N | N |
|  |  |  | 3105 | N | N | N | N | N | N | N | N | N | N | N | N | N | N |
|  |  |  | 3106 | N | N | N | N | N | N | N | N | N | N | N | N | N | N |
|  |  | 150 | 3201 | N | N | N | N | N | N | N | N | N | N | N | N | N | N |
|  |  |  | 3202 | N | N | N | N | N | N | N | N | N | N | N | N | N | N |
|  |  |  | 3203 | N | N | N | N | N | N | N | N | N | N | N | N | N | N |
|  |  |  | 3204 | N | N | N | N | N | N | N | N | N | N | N | N | N | N |
|  |  |  | 3205 | N | N | N | N | N | N | N | N | N | N | N | N | N | N |
|  |  |  | 3206 | N | N | N | N | N | N | N | N | N | N | N | N | N | N |
|  |  | 300 | 3301 | N | N | N | N | N | N | N | N | N | N | N | N | N | N |
|  |  |  | 3302 | N | N | N | N | N | N | N | N | N | N | N | N | N | N |
|  |  |  | 3303 | N | N | N | N | N | N | N | N | N | N | N | N | N | N |
|  |  |  | 3304 | N | N | N | N | N | N | N | N | N | N | N | N | N | N |
|  |  |  | 3305 | N | N | N | N | N | N | N | N | N | N | N | N | N | N |
|  |  |  | 3306 | N | N | N | N | N | N | N | N | N | N | N | N | N | N |
|  |  |  | 3307 | N | N | N | N | N | N | N | N | N | N | N | N | N | N |
|  |  |  | 3308 | N | N | N | N | N | N | N | N | N | N | N | N | N | N |
|  | ENU | 100 | 3401 | N | | N  N | | N  N | | N | | N | | N (Sacrificed) | | - | |
|  |  |  | 3402 | N | | N  N | | N  N | | N | | N | | N (Sacrificed) | | - | |
|  |  |  | 3403 | N | | N  N | | N  N | | N | | N | | N (Sacrificed) | | - | |
|  |  |  | 3404 | N | | N  N | | N  N | | N | | N | | N (Sacrificed) | | - | |
|  |  |  | 3405 | N | | N  N | | N  N | | N | | N | | N (Sacrificed) | | - | |
|  |  |  | 3406 | N | | N  N | | N  N | | N | | N | | N (Sacrificed) | | - | |

|  |  |  |  | Day of experiment | | | | | | | | | | | | | |
| --- | --- | --- | --- | --- | --- | --- | --- | --- | --- | --- | --- | --- | --- | --- | --- | --- | --- |
|  | Substance | Dose (mg/kg/day, p.o.) | Animal ID No. | 15 | | 16 | | 17 | | 18 | | 19 | | 20 | | 21 | |
|  |  |  |  | 1st | 2nd | 1st | 2nd | 1st | 2nd | 1st | 2nd | 1st | 2nd | 1st | 2nd | 1st | 2nd |
|  | 0.5% MC | 0 | 3001 | N | N | N | N | N | N | N | N | N | N | N | N | N | N |
|  |  |  | 3002 | N | N | N | N | N | N | N | N | N | N | N | N | N | N |
|  |  |  | 3003 | N | N | N | N | N | N | N | N | N | N | N | N | N | N |
|  |  |  | 3004 | N | N | N | N | N | N | N | N | N | N | N | N | N | N |
|  |  |  | 3005 | N | N | N | N | N | N | N | N | N | N | N | N | N | N |
|  |  |  | 3006 | N | N | N | N | N | N | N | N | N | N | N | N | N | N |
|  | TBHP | 75 | 3101 | N | N | N | N | N | N | N | N | N | N | N | N | N | N |
|  |  |  | 3102 | N | N | N | N | N | N | N | N | N | N | N | N | N | N |
|  |  |  | 3103 | N | N | N | N | N | N | N | N | N | N | N | N | N | N |
|  |  |  | 3104 | N | N | N | N | N | N | N | N | N | N | N | N | N | N |
|  |  |  | 3105 | N | N | N | N | N | N | N | N | N | N | N | N | N | N |
|  |  |  | 3106 | N | N | N | N | N | N | N | N | N | N | N | N | N | N |
|  |  | 150 | 3201 | N | N | N | N | N | N | N | N | N | N | N | N | N | N |
|  |  |  | 3202 | N | N | N | N | N | N | N | N | N | N | N | N | N | N |
|  |  |  | 3203 | N | N | N | N | N | N | N | N | N | N | N | N | N | N |
|  |  |  | 3204 | N | N | N | N | N | N | N | N | N | N | N | N | N | N |
|  |  |  | 3205 | N | N | N | N | N | N | N | N | N | N | N | N | N | N |
|  |  |  | 3206 | N | N | N | N | N | N | N | N | N | N | N | N | N | N |
|  |  | 300 | 3301 | N | N | N | N | N | N | N | N | N | N | N | N | N | N |
|  |  |  | 3302 | N | N | N | N | N | N | N | N | N | N | N | N | N | N |
|  |  |  | 3303 | N | N | N | N | N | N | N | N | N | N | N | N | N | N |
|  |  |  | 3304 | N | N | N | N | N | N | N | N | N | N | N | N | N | N |
|  |  |  | 3305 | N | N | N | N | N | N | N | N | N | N | N | N | N | N |
|  |  |  | 3306 | N | N | N | N | N | N | N | N | N | N | N | N | N | N |
|  |  |  | 3307 | N | N | N | N | N | N | N | N | N | N | N | N | N | N |
|  |  |  | 3308 | N | N | N | N | N | N | N | N | N | N | N | N | N | N |
|  | ENU | 100 | 3401 | - | | -  N | | -  N | | - | | - | | - | | - | |
|  |  |  | 3402 | - | | -  N | | -  N | | - | | - | | - | | - | |
|  |  |  | 3403 | - | | -  N | | -  N | | - | | - | | - | | - | |
|  |  |  | 3404 | - | | -  N | | -  N | | - | | - | | - | | - | |
|  |  |  | 3405 | - | | -  N | | -  N | | - | | - | | - | | - | |
|  |  |  | 3406 | - | | -  N | | -  N | | - | | - | | - | | - | |

|  |  |  |  | Day of experiment | | | | | | | | | | | | | |
| --- | --- | --- | --- | --- | --- | --- | --- | --- | --- | --- | --- | --- | --- | --- | --- | --- | --- |
|  | Substance | Dose (mg/kg/day, p.o.) | Animal ID No. | 22 | | 23 | | 24 | | 25 | | 26 | | 27 | | 28 | |
|  |  |  |  | 1st | 2nd | 1st | 2nd | 1st | 2nd | 1st | 2nd | 1st | 2nd | 1st | 2nd | 1st | 2nd |
|  | 0.5% MC | 0 | 3001 | N | N | N | N | N | N | N | N | N | N | N | N | N | N |
|  |  |  | 3002 | N | N | N | N | N | N | N | N | N | N | N | N | N | N |
|  |  |  | 3003 | N | N | N | N | N | N | N | N | N | N | N | N | N | N |
|  |  |  | 3004 | N | N | N | N | N | N | N | N | N | N | N | N | N | N |
|  |  |  | 3005 | N | N | N | N | N | N | N | N | N | N | N | N | N | N |
|  |  |  | 3006 | N | N | N | N | N | N | N | N | N | N | N | N | N | N |
|  | TBHP | 75 | 3101 | N | N | N | N | N | N | N | N | N | N | N | N | N | N |
|  |  |  | 3102 | N | N | N | N | N | N | N | N | N | N | N | N | N | N |
|  |  |  | 3103 | N | N | N | N | N | N | N | N | N | N | N | N | N | N |
|  |  |  | 3104 | N | N | N | N | N | N | N | N | N | N | N | N | N | N |
|  |  |  | 3105 | N | N | N | N | N | N | N | N | N | N | N | N | N | N |
|  |  |  | 3106 | N | N | N | N | N | N | N | N | N | N | N | N | N | N |
|  |  | 150 | 3201 | N | ILA | ILA | ILA | ILA | ILA | ILA | ILA | ILA | ILA | ILA | ILA | ILA | ILA |
|  |  |  | 3202 | N | ILA | ILA | ILA | ILA | ILA | ILA | ILA | ILA | ILA | ILA | ILA | ILA | ILA |
|  |  |  | 3203 | N | ILA | ILA | ILA | ILA | ILA | ILA | ILA | ILA | ILA | ILA | ILA | ILA | ILA |
|  |  |  | 3204 | N | ILA | ILA | ILA | ILA | ILA | ILA | ILA | ILA | ILA | ILA | ILA | ILA | ILA |
|  |  |  | 3205 | N | ILA | ILA | ILA | ILA | ILA | ILA | ILA | ILA | ILA | ILA | ILA | ILA | ILA |
|  |  |  | 3206 | N | ILA | ILA | ILA | ILA | ILA | ILA | ILA | ILA | ILA | ILA | ILA | ILA | ILA |
|  |  | 300 | 3301 | N | ILA | ILA | ILA | ILA | ILA | ILA | ILA | ILA | ILA | ILA | ILA | ILA | ILA |
|  |  |  | 3302 | N | ILA | ILA | ILA | ILA | ILA | ILA | ILA | ILA | ILA | ILA | ILA | ILA | ILA |
|  |  |  | 3303 | N | ILA | ILA | ILA | ILA | ILA | ILA | ILA | ILA | ILA | ILA | ILA | ILA | ILA |
|  |  |  | 3304 | N | ILA | ILA | ILA | ILA | ILA | ILA | ILA | ILA | ILA | ILA | ILA | ILA | ILA |
|  |  |  | 3305 | N | ILA | ILA | ILA | ILA | ILA | ILA | ILA | ILA | ILA | ILA | ILA | ILA | ILA |
|  |  |  | 3306 | N | ILA | ILA | ILA | ILA | ILA | ILA | ILA | ILA | ILA | ILA | ILA | ILA | ILA |
|  |  |  | 3307 | N | ILA | ILA | ILA | ILA | ILA | ILA | ILA | ILA | ILA | ILA | ILA | ILA | ILA |
|  |  |  | 3308 | N | ILA | ILA | ILA | ILA | ILA | ILA | ILA | ILA | ILA | ILA | ILA | ILA | ILA |
|  | ENU | 100 | 3401 | - | | -  N | | -  N | | - | | - | | - | | - | |
|  |  |  | 3402 | - | | -  N | | -  N | | - | | - | | - | | - | |
|  |  |  | 3403 | - | | -  N | | -  N | | - | | - | | - | | - | |
|  |  |  | 3404 | - | | -  N | | -  N | | - | | - | | - | | - | |
|  |  |  | 3405 | - | | -  N | | -  N | | - | | - | | - | | - | |
|  |  |  | 3406 | - | | -  N | | -  N | | - | | - | | - | | - | |

|  |  |  |  | Day of experiment | | |
| --- | --- | --- | --- | --- | --- | --- |
|  | Substance | Dose (mg/kg/day, p.o.) | Animal ID No. | 29 | 30 | 31 |
|  |  |  |  | 2nd | 2nd | (Sacrificed)  2nd |
|  | 0.5% MC | 0 | 3001 | N  N | N  N | N |
|  |  |  | 3002 | N  N | N  N | N |
|  |  |  | 3003 | N  N | N  N | N |
|  |  |  | 3004 | N  N | N  N | N |
|  |  |  | 3005 | N  N | N  N | N |
|  |  |  | 3006 | N  N | N  N | N |
|  | TBHP | 75 | 3101 | N  N | N  N | N |
|  |  |  | 3102 | N  N | N  N | N |
|  |  |  | 3103 | N  N | N  N | N |
|  |  |  | 3104 | N  N | N  N | N |
|  |  |  | 3105 | N  N | N  N | N |
|  |  |  | 3106 | N  N | N  N | N |
|  |  | 150 | 3201 | ILA | N | N |
|  |  |  | 3202 | ILA | N | N |
|  |  |  | 3203 | ILA | N | N |
|  |  |  | 3204 | ILA | N | N |
|  |  |  | 3205 | ILA | N | N |
|  |  |  | 3206 | ILA | N | N |
|  |  | 300 | 3301 | ILA | N | N |
|  |  |  | 3302 | ILA | ILA | N |
|  |  |  | 3303 | ILA | ILA | N |
|  |  |  | 3304 | ILA | N | N |
|  |  |  | 3305 | ILA | N | N |
|  |  |  | 3306 | ILA | ILA | ILA |
|  |  |  | 3307 | ILA | N | N |
|  |  |  | 3308 | ILA | N | N |
|  | ENU | 100 | 3401 | - | -  N | -  N |
|  |  |  | 3402 | - | -  N | -  N |
|  |  |  | 3403 | - | -  N | -  N |
|  |  |  | 3404 | - | -  N | -  N |
|  |  |  | 3405 | - | -  N | -  N |
|  |  |  | 3406 | - | -  N | -  N |

0.5% MC: Negative control (0.5 w/v% Methylcellulose solution, 10 mL/kg).

ENU: Positive control (*N* -ethyl-*N* -nitrosourea, 10 mL/kg, dose once a day, for 2 days, expression period; 10 days).

1st: Just before the administration, 2nd: After the administration.

N: Normal, ILA: Increase in locomotor activity.

Supplementary table 2. Historical data of negative controls (Transgenic rodent gene mutation assay (*lacZ* assay))

| Group | n | Mutant frequency [×10^−6^] (Mean ± S.D.) | Acceptable range * | |
| --- | --- | --- | --- | --- |
|  |  |  | Lower | Upper |
| [MutaMouse: male; liver] Negative control | 122 | 43.1 ± 13.5 | 16.6 | 69.6 |
| [MutaMouse: male; stomach] Negative control | 107 | 43.8 ± 12.8 | 18.7 | 68.9 |

The historical control data presented above was compiled from October 10, 2014 to November 1, 2019.

Negative control: Including water for injection, 0.5% methylcellulose, corn oil, *etc*.

* The confidence interval for negative control is 95%.
